# Digital public services and subjective well-being in rural China: health and multidomain lifestyle pathways in the cross-provincial Dabie Mountains region

**DOI:** 10.3389/fpubh.2026.1884928

**Published:** 2026-08-27

**Authors:** Lei Jiang, Yuqiao Ma, Zihan Yang, Hao Li

**Affiliations:** 1 School of Business, Xinyang Normal University, Xinyang, Henan, China; 2 Research Institute of the Economic and Social Development in the Dabie Mountains, Xinyang, Henan, China

**Keywords:** digital health, digital public services, food-purchasing access, physical activity, stress management, subjective well-being

## Abstract

**Introduction:**

Against the backdrop of China’s Digital Rural Development Strategy and the growing emphasis on integrated health, food-purchasing access, and lifestyle approaches to well-being, digital public services (DPS) are increasingly important for rural public health. However, little is known about the association between integrated DPS and subjective well-being (SWB) in geographically constrained mountainous regions or about the health- and lifestyle-related pathways underlying this association.

**Methods:**

Using survey data from 1,287 rural residents across 26 counties in the Dabie Mountains Region, this study constructs comprehensive DPS and SWB indices using the entropy weight method and employs ordinary least squares as the baseline estimator, complemented by robustness checks and an instrumental-variable sensitivity analysis.

**Results:**

The results show that DPS is positively associated with SWB, and the association remains stable across alternative specifications. The estimated coefficients are descriptively larger in the Anhui section, among low-income households, and among residents with lower education levels. Separate exploratory models yield evidence consistent with five potential indirect pathways: livelihood stability and income diversification, improved education and medical service quality, enriched cultural and social interaction, greater daily-life purchasing and delivery convenience, and improved integrated health and multidomain lifestyle behaviors, including physical activity, sleep quality, stress management, and digital health-tool use.

**Discussion:**

These findings suggest that DPS may support rural well-being through service access, digital purchasing and delivery functions, and technology-supported multidomain health management, providing context-specific empirical evidence relevant to the design of integrated digital public service strategies in mountainous communities.

## 1 Introduction

Subjective well-being (SWB) is a core indicator for evaluating development quality and livelihood improvement in a country or region. It also reflects the government’s commitment to public welfare (1). Data from the China Household Finance Survey show that the share of satisfied households in China rose from 56.7% in 2013 to 70.2% in 2017, suggesting an overall improvement in well-being (2). However, China still ranks relatively low in global happiness assessments. According to the World Happiness Report 2026, China ranks 65th among 147 countries, indicating continued scope for improving population well-being (3). China has a rural population of over 400 million. Rural areas have long been constrained by geographic barriers and urban–rural gaps, which are associated with lower well-being among rural residents (4). In mountainous communities, geographic isolation can also restrict access to health information, everyday food-purchasing and delivery services, and opportunities to sustain regular physical activity, healthy sleep, and effective stress management.

Against the national strategy of Digital Rural Development and comprehensive rural revitalization, the digital transformation of public services has become a key approach to narrowing the rural–urban service gap, optimizing resource allocation, and supporting rural well-being (5). The 2024 No. 1 Central Document highlights the use of digital technology to upgrade rural public services and enhance farmers’ sense of gain, underscoring the policy priority of linking digitalization to rural well-being. Digital public services (DPS) integrate digital technology with education, medical care, government services, and daily-life services. They can reduce spatial and temporal barriers, lower service-access costs, and support more equitable access to basic public services (6). Within this integrated system, digital education and telemedicine can deliver health information and self-management support; digital platforms can support physical activity, sleep, and stress management; and rural e-commerce, express delivery, and online meal-ordering services can improve the convenience and geographic reach of everyday food purchasing. Nevertheless, mountainous and cross-provincial rural regions still face weak digital infrastructure, unbalanced service supply, and low utilization rates, which may limit the well-being benefits associated with DPS (7).

The Dabie Mountains Region is a typical cross-provincial mountainous area in central China, covering 26 counties in Anhui, Hubei, and Henan provinces. As an important watershed between the Yangtze and Huaihe rivers and a key old revolutionary base area, the region features widespread hills and mountains, scattered rural settlements, relatively insufficient digital infrastructure, and unbalanced basic public service supply (8). Unlike single-province samples in previous studies, the Dabie Mountains Region shares similar topographic constraints but differs in provincial policy support and digital development levels. Such features provide a valuable empirical setting for examining the association between DPS and rural residents’ SWB, as well as heterogeneous association patterns and potential indirect pathways. At present, few studies focus on the DPS-SWB nexus in cross-provincial mountainous areas, leaving limited evidence for policy design in the Dabie Mountains Region.

Existing research on digital public services and rural residents’ SWB has grown rapidly in recent years, but notable research gaps remain at both the international and Chinese contextual levels. Internationally, empirical studies have explored the welfare associations of digital public services in rural settings, yet most are based on single-country samples (9, 10). Few studies have examined the unique challenges of cross-administrative mountainous regions, where policy fragmentation and topographic constraints give rise to distinct digital divide patterns that differ fundamentally from plain or urban areas (11, 12). Furthermore, recent empirical studies and reviews have documented substantial contextual heterogeneity in the welfare associations of digital services, indicating that findings from one regional setting cannot be readily generalized to others and underscoring the need for context-specific empirical evidence (6, 13). In particular, research on inclusive digital design has highlighted the potential of user-centric service features to reduce well-being inequalities among disadvantaged rural groups, but empirical evidence from mountainous low-literacy contexts remains scarce (14, 15). Few studies have examined how digital public services are associated with well-being through health-management, food-purchasing access, and complementary lifestyle pathways in resource-constrained rural settings.

Within the Chinese context, existing studies face three more specific limitations that align with these global gaps and further constrain the reliability and generalizability of findings. First, most studies focus on single provinces or geographically homogeneous regions, with limited empirical evidence from cross-provincial mountainous areas (16). Such narrow sampling limits the external validity of conclusions and makes it difficult to extend findings to other mountainous regions with similar topographic and institutional features. Second, many studies treat digital technology and public services as separate analytical factors rather than examining them as an integrated DPS system (5). Most research investigates either public service supply or digital technology adoption in isolation, failing to capture their combined well-being associations. Meanwhile, the measurement of DPS remains oversimplified, often relying on single indicators such as internet penetration or overall service satisfaction, which cannot fully reflect the multidimensional structure of integrated digital public services. Third, many studies do not sufficiently address potential confounding and reverse association (17). Most importantly, existing research rarely identifies intermediate mechanisms linking digital public services to rural residents’ well-being through health-related behaviors and complementary lifestyle factors such as physical activity, sleep quality, and stress regulation. The role of digital shopping, express delivery, and online meal ordering in reducing geographic barriers to everyday food purchasing also remains underexplored. These gaps limit understanding of how integrated digital services can support multidomain public health strategies in resource-constrained communities.

To address these gaps, this study focuses on rural residents in the Dabie Mountains Region spanning Anhui, Hubei, and Henan provinces. The analysis uses a multistage stratified sample of 1,287 rural residents across 26 counties. First, the study constructs comprehensive DPS and SWB indices using the entropy weight method and examines their association and heterogeneity across provinces, income groups, and education levels. Second, the DPS index integrates digital education, medical care, government services, and daily-life services, including rural e-commerce and respondents’ use of digital payment, online shopping, express delivery, and meal-ordering functions. Third, five potential indirect pathways are examined: livelihood stability and income diversification, improved education and medical service quality, enriched cultural and social interaction, greater daily-life purchasing and delivery convenience, and integrated health and lifestyle behaviors. Fourth, OLS is used as the baseline estimator, Ordered Probit is used as a robustness check, and average county-level terrain slope is used in an instrumental-variable sensitivity analysis. Because the exclusion restriction cannot be verified directly, the IV results are interpreted as complementary evidence rather than definitive causal estimates.

This study makes three theoretical contributions, alongside context-specific empirical contributions focused on an understudied cross-provincial mountainous region. First, the results extend the infrastructure endowment perspective by indicating that the association between DPS and well-being varies not only with headline infrastructure coverage but also with intergovernmental policy support and the depth of infrastructure penetration in dispersed villages (18). Second, the results add nuance to the digital-literacy threshold perspective by showing descriptively larger DPS–SWB coefficients among residents with lower education levels, a pattern potentially consistent with inclusive service design and assisted access (19). Third, the mechanism analysis extends SWB theory by showing that socio-cultural connection is a prominent pathway in geographically isolated settlements, while integrated health and lifestyle behaviors provide a complementary pathway alongside livelihood resilience, service quality, and daily-life purchasing and delivery convenience (20). By incorporating physical activity, sleep, stress management, digital health use, and food-purchasing convenience into the mechanism framework, the study links digital public service research with multidomain lifestyle approaches to well-being. Empirically, the study constructs a multidimensional DPS index integrating objective supply data and subjective user experience, reports baseline associations together with robustness checks and complementary IV estimates, and documents heterogeneous coefficient patterns across provincial sections, income groups, and education levels. This design is distinct from the related study by Li et al. (17), which used a Henan-only sample and modelled digital technology development and public service provision as separate constructs. The present study uses a cross-provincial mountainous sample, an integrated DPS index, and a health and multidomain lifestyle pathway framework.

The remainder of this paper is structured as follows. Section 2 reviews the theoretical foundation and develops research hypotheses. Section 3 introduces the research design, including data sources, variable definitions, and model specifications. Section 4 reports the baseline association, robustness checks, instrumental-variable sensitivity analysis, heterogeneity analysis, and exploratory mediation results. Section 5 presents the discussion, conclusions, and policy implications.

## 2 Potential mechanisms and research hypotheses

The integration of digital technology and public services has become an important component of rural service delivery. This study takes DPS as the core explanatory variable. DPS comprise four functional dimensions: digital education, digital medical care, digital government services, and digital life services (21). They combine objective service supply with subjective usage experience. This section explains the pathways through which DPS may be associated with the SWB of rural residents and develops the corresponding hypotheses.

### 2.1 Direct association between DPS and rural residents’ SWB

DPS represent the digital transformation of conventional public services. They can reduce spatial and temporal constraints on service access and may help address insufficient access, uneven distribution, and low service quality in rural areas (6). Three potential pathways may underlie the direct association between DPS and SWB.

#### 2.1.1 Improved accessibility reduces resource acquisition costs

Traditional rural public services are often restricted by geographic conditions. Residents in remote areas must travel long distances to visit county hospitals or government service centers, which creates high time and economic costs (17). DPS may help mitigate these costs through digital empowerment. In digital medical care, telemedicine equipment in village clinics allows residents to receive professional advice without traveling to urban areas. In digital government services, online platforms support social security payment, document handling, and policy inquiry without repeated trips to government offices (22). Lower costs of accessing key resources may improve life quality and satisfaction and thereby be associated with higher SWB (23). This cost-reduction mechanism provides the first potential channel underlying Hypothesis 1.

#### 2.1.2 Balanced resource allocation alleviates inequality

Uneven distribution of high-quality educational and medical resources is a major constraint on rural SWB (17). DPS can support the cross-regional sharing of high-quality resources. In digital education, online platforms connect rural schools with high-quality urban educational resources, enabling students in remote areas to access advanced teaching content (23). In digital medical care, telemedicine systems support cooperation between county hospitals and village clinics, helping rural residents obtain basic medical services locally (24). Such resource equalization may reduce perceived deprivation and support higher SWB. This resource-equalization mechanism provides the second potential channel of Hypothesis 1.

#### 2.1.3 Personalized services match diverse resident needs

Rural residents have diverse demands for public services. Older adult residents focus on medical accessibility, farming households care about agricultural technical support, and left-behind children need educational assistance. DPS can support intelligent matching to meet diverse demands. Digital life platforms provide personalized recommendations for daily supplies and production materials (25). Digital government platforms push targeted policies such as farming subsidies and skills training to different groups (26). This demand-oriented service model may enhance residents’ sense of gain and be associated with higher SWB. This personalized service mechanism provides the third potential channel proposed in Hypothesis 1.

Based on the above analysis, this study proposes Hypothesis 1 (H1): DPS is significantly and positively associated with the SWB of rural residents.

This hypothesis will be evaluated using baseline OLS regression in Section 4.1, with robustness assessed in Section 4.2.

### 2.2 Indirect pathways linking DPS to rural residents’ SWB

DPS may be associated with SWB both directly and indirectly through changes in key dimensions of rural life. This study examines five potential pathways.

#### 2.2.1 Mediating path 1: livelihood stability and income diversification

DPS may be associated with greater livelihood stability and more diversified income structures among rural residents through several economic mechanisms. DPS may reduce the overall cost of agricultural production and daily management (27). Residents can access low-cost production materials, standardized planting and breeding guidance, and intelligent early warning systems for crop diseases, all of which reduce material, labor, and time inputs. Meanwhile, digital platforms break geographic limits, link rural producers with broader consumer markets, and shorten intermediate circulation links, which may help improve pricing power and increase net income from agricultural management (28). In addition, DPS provide timely access to employment information, entrepreneurial support policies, and practical skills training, which may create more stable off-farm employment opportunities and raise wage income (29). Data-driven information may also help residents grasp market demand, price fluctuations, and supporting policies, supporting more reasonable production decisions, lower operational risks, and more stable income expectations (30). Greater livelihood stability, more diversified income sources, and more predictable income expectations are important predictors of SWB beyond absolute income levels. Reduced income volatility and expanded non-farm employment opportunities may strengthen residents’ sense of economic security and life satisfaction and thereby be associated with higher SWB. This provides the first potential indirect pathway in Hypothesis 2.

#### 2.2.2 Mediating path 2: the quality of education and medical services

DPS may help overcome long-standing geographic barriers that restrict access to high-quality education and medical resources in rural areas, providing a more stable foundation for well-being. In education, digital platforms can support continuous online training for rural teachers and improve local teaching capacity. Cross-regional digital courses and synchronized teaching resources may also enable rural students to access educational content that was previously concentrated in cities (31, 32). In medical care, telemedicine systems can connect rural clinics with urban and county-level hospitals, allowing residents to obtain professional consultations without long-distance travel (23). Digital health-management tools can provide reminders for disease prevention, health monitoring, and appropriate service use (33). Better educational and health-service quality may strengthen human capital and perceived health security, both of which are associated with SWB. This provides the second potential indirect pathway in Hypothesis 2.

#### 2.2.3 Mediating path 3: cultural life and social interactions

DPS also enrich rural cultural life and strengthen social connections, both of which support higher SWB. Digital cultural channels provide diverse spiritual and cultural products such as online exhibitions, folk art displays, and local cultural live broadcasts (34). These services may alleviate cultural shortages and reduce isolation among left-behind older adult individuals and women. Digital platforms including village affairs groups, online mutual-aid channels, and community-based online activities promote more frequent and smoother communication among residents. These platforms may enhance trust and community cohesion, reduce loneliness, and foster a stronger sense of belonging and identity (35). Improved cultural satisfaction and social integration bring more positive emotions and higher life evaluations, further supporting steady gains in SWB. This provides the third potential indirect pathway in Hypothesis 2.

#### 2.2.4 Mediating path 4: daily-life purchasing and delivery convenience

DPS integrate various daily service functions into unified digital platforms, improving the efficiency and convenience of rural daily life. Residents can complete online shopping, express delivery, takeout or meal ordering, fee payment, and certificate applications through rural e-commerce stations or mobile applications, which may reduce the time and effort required for routine transactions (36). These functions are particularly relevant in dispersed mountainous communities, where limited local retail options and long travel distances can constrain everyday purchasing. Older adult residents who are less familiar with digital operations can obtain on-site guidance from village digital assistants, while farming households can purchase production materials and market agricultural products through the same service networks. Greater daily-life purchasing and delivery convenience may reduce the time and travel required to obtain everyday goods, including food, and may support life satisfaction by lowering transaction burdens in dispersed mountainous communities (37). Broader access to food-purchasing channels may reduce geographic constraints on everyday food acquisition, expand purchasing options, and improve the convenience of obtaining food in dispersed mountainous communities (38). This provides the fourth potential indirect pathway proposed in Hypothesis 2.

#### 2.2.5 Mediating path 5: integrated health and lifestyle behaviors

DPS may support rural residents’ SWB by facilitating a cluster of complementary health and lifestyle behaviors. Digital education and telemedicine platforms can provide health information, chronic-disease self-management support, exercise guidance, sleep-management advice, and stress-management resources. Physical activity, sleep, and stress management do not operate in isolation. Regular activity can support sleep and emotional regulation, while adequate sleep and effective stress management can strengthen residents’ capacity to maintain healthy daily routines (39). Digital health applications and village-level terminals can provide repeated reminders, self-monitoring functions, and access to professional services (33). Accordingly, residents with greater access to and use of DPS may report better health-information use, health self-management, physical activity, sleep, stress-management practices, and digital health-tool use. These behaviors are associated with physical functioning, emotional balance, and more favorable evaluations of life, providing a theoretically coherent pathway from integrated digital services to SWB.

Based on the above analysis, this study proposes Hypothesis 2 (H2):

The positive association between DPS and rural residents’ SWB is expected to be statistically linked to five potential indirect pathways: livelihood stability and income diversification, improved education and medical service quality, enriched cultural and social interaction, greater daily-life purchasing and delivery convenience, and improved health and multidomain lifestyle behaviors.

### 2.3 Heterogeneous associations between DPS and rural residents’ SWB

The association between DPS and SWB may vary with regional conditions and individual characteristics, which shape access to and use of DPS. Based on the characteristics of the Dabie Mountains Region, this study examines heterogeneity across DPS subdimensions, provincial sections, income groups, and education levels.

#### 2.3.1 Heterogeneity across DPS sub-dimensions

According to Maslow’s hierarchy of needs, different DPS subdimensions correspond to different types of need, and their associations with SWB may vary with demand urgency (40). Digital medical care and digital education address basic health, safety, and human-capital needs, which may be especially salient in remote rural areas with constrained access to hospitals and high-quality schools. Telemedicine can reduce the need for long-distance travel, while digital education can expand access to teaching resources (4). By contrast, digital government and life services primarily improve transaction efficiency and convenience and may therefore show smaller associations with SWB (41).

#### 2.3.2 Regional heterogeneity: the moderating role of infrastructure endowment

From an infrastructure endowment perspective, digital infrastructure provides an important foundation for DPS (18). Better infrastructure may lower access costs and strengthen the association between DPS and SWB. The Dabie Mountains Region spans Anhui, Hubei, and Henan provinces and exhibits uneven digital development and marked topographic constraints. The Anhui section has relatively complete digital infrastructure, including broad 5G coverage and dense rural e-commerce stations. The Hubei section has moderate infrastructure supported by telemedicine and digital-rural programs, whereas the Henan section faces scattered settlements and more limited investment. These differences may shape service use: deeper infrastructure penetration in Anhui can support broader use, while weak signals and insufficient service outlets may constrain use in Henan (42). The expected pattern is therefore a stronger DPS–SWB association where digital infrastructure and institutional support are more developed.

#### 2.3.3 Income heterogeneity: guidance from need hierarchy theory

Guided by the extended Maslow’s Need Hierarchy Theory, income levels shape demand priorities (40). Low-income households face tight constraints in meeting basic needs, with large spending on medical care and education. Digital medical care and free digital education services may ease economic pressure and therefore show greater well-being relevance for low-income households. Middle- and high-income households pay more attention to efficiency. They may place greater value on digital government and life services associated with efficiency, life quality, and achievement (43).

#### 2.3.4 Education heterogeneity: constraints of digital literacy

From a digital-literacy threshold perspective, education levels shape individuals’ capacity to use digital tools, with lower education typically corresponding to greater digital-adoption barriers (42, 44). However, inclusive design features can mitigate these barriers, particularly for older adults and rural populations with limited digital literacy (15, 45). In the rural Dabie Mountains Region, dialect voice navigation, simplified interfaces, and village digital assistants may help residents with lower education use digital medical and public services and narrow information gaps, producing a possible “catch-up” pattern in SWB (22). Residents with higher education often have higher service expectations that may exceed the functions currently provided by basic DPS, potentially creating an expectation gap and a smaller observed association with SWB (23).

Based on the above analysis, this study proposes Hypothesis 3 (H3) and its sub-hypotheses:

*H3a:* Digital medical care and digital education have stronger positive associations with SWB than digital government services and digital life services.

This expected coefficient pattern will be examined through sub-dimensional regressions in Section 4.4.1, with differences in coefficient magnitude interpreted descriptively.

*H3b:* The association between DPS and SWB varies across provincial sections of the Dabie Mountains Region.

*H3c:* The positive association between DPS and SWB is stronger for low-income groups than for middle- and high-income groups.

*H3d:* The positive association between DPS and SWB is stronger for low-education groups than for high-education groups, a pattern potentially consistent with inclusive service design and assisted access.

The coefficient patterns proposed in H3b–H3d will be examined through provincial, income-based, and education-based subsample regressions in Section 4.4.

## 3 Research design

### 3.1 Model construction

This study uses a multistep econometric framework to examine the association between DPS and rural residents’ SWB and to evaluate potential indirect pathways. The SWB measure follows the tripartite conceptualization of subjective well-being proposed by Diener et al. (46), encompassing life satisfaction, positive affect, and negative affect, with context-specific items adapted to the rural Chinese setting. OLS is used as the baseline estimator. As a supplementary robustness check, the continuous SWB measure is also converted into ordered categories and estimated using an Ordered Probit model.

#### 3.1.1 Baseline regression model: association test

The dependent variable SWB is a continuous composite index ranging from 0 to 1. OLS provides a straightforward estimate of conditional mean associations, while county-clustered standard errors and alternative specifications are used to assess robustness. The baseline model is specified as follows (Equation 1):


$$SWB_{i}=\alpha_{0}+\alpha_{1}DPS_{i}+\alpha_{2}X_{i}+\varepsilon_{i}$$ (1)

where SWB denotes the SWB of resident *i*; DPS represents the level of DPS; X is a vector of control variables including individual, household, and county characteristics; and ε is the random error term.

#### 3.1.2 Exploratory mediation model

To examine the five potential indirect pathways linking DPS to SWB, this study uses a two-step OLS mediation framework. The models estimate whether DPS is associated with each mediator and whether the mediator is associated with SWB after controlling for DPS and the full set of covariates. Given the cross-sectional design, these estimates are interpreted as statistical indirect associations rather than proof of temporal causation. The model is specified in two steps.

Step 1: The association between DPS and the mediating variable $M_{ij}$ _:_


$$M_{i}=\beta_{0}+\beta_{1}DPS_{i}+\beta_{2}X_{i}+\varepsilon_{i}$$ (2)

Step 2: The conditional associations of DPS and the mediating variable with SWB.


$$SWB_{i}=\delta_{0}+\delta_{1}DPS_{i}+\delta_{2}M_{i}+\delta_{3}X_{i}+\varepsilon_{i}$$ (3)

A statistically significant coefficient in Step 1 indicates that DPS is associated with the mediator. Jointly significant coefficients for DPS and the mediator in Step 2, together with a bootstrap confidence interval for the coefficient product that excludes zero, support the presence of an indirect association.

### 3.2 Data collection

The survey was conducted between February and September 2024. A multistage stratified sampling design was used to improve representation across provincial sections and areas with different levels of economic and digital development. According to geographic and administrative divisions, the Dabie Mountains Region is divided into three provincial sub-regions: the Anhui section, represented by Lu’an and Anqing, which features relatively complete digital infrastructure and rich cultural and tourism resources; the Hubei section, represented by Huanggang and Xiaogan, which concentrates red cultural resources and characteristic agricultural industries; and the Henan section, represented by Xinyang and Nanyang, which is dominated by low hills and scattered rural settlements (8). The sampling procedure follows a multi-stage framework: province-county-township-village-resident.

Specifically, 8 to 9 counties were selected from each province, including both economically developed counties and less developed counties with typical mountainous features. In each county, 2 to 3 townships were chosen to balance areas with high and low levels of digital infrastructure and agricultural modernization. In each township, 2 to 3 key villages were selected, including grain-producing villages, cash-crop villages, suburban villages, and remote mountain villages. In each village, 8 to 10 rural residents were randomly interviewed.

Participants were eligible if they were aged between 18 and 65 years, had resided in the village for at least 1 year, and were not temporary migrant workers. The age range reflected the predefined sampling frame used in the survey. Accordingly, the findings should not be generalized to rural residents older than 65 years. These criteria were intended to ensure that respondents could clearly express their subjective perceptions and had sufficient experience with local public services. A total of 1,600 questionnaires were distributed across the three provincial sections of the Dabie Mountains Region.

After data cleaning, the final analytic sample included 1,287 observations, representing an effective response rate of 80.4%. Of the 313 excluded questionnaires, 186 contained sufficient observable information for comparison with the analytic sample, whereas 127 unreturned forms contained no respondent-level data. Table 1 therefore compares the observable characteristics of the 186 completed invalid responses with those of the 1,287 valid responses.

**Table 1:** Comparison of observable characteristics between completed invalid and valid responses.

Characteristic	Completed invalid responses (*n* = 186)	Valid Responses (*n* = 1,287)	*t*-value	*p*-value
Gender (male ratio, %)	51.3	50.1	0.31	0.757
Age category (mean)	2.71	2.81	0.49	0.624
Years of schooling (mean)	5.98	5.78	0.71	0.478
Per capita income (10 k RMB, mean)	2.08	2.32	0.81	0.418
Marital status (married ratio, %)	85.7	81.3	1.58	0.114

The *t*-tests compare observable characteristics between completed but invalid responses and valid responses to assess potential response bias. Unreturned questionnaires (*n* = 127) are not included in the comparison as no characteristic data are available. All *p*-values are above 0.10, indicating no statistically significant difference in observable characteristics between the two groups. Age was coded as 1, 18–30 years; 2, 31–40 years; 3, 41–50 years; 4, 51–60 years; and 5, 61–65 years. Education was coded as 1, primary school or below; 2, junior middle school; 3, senior secondary school/vocational school; 4, junior college, and 5, bachelor’s degree or above.

To reduce limitations associated with reliance on survey data alone, this study integrates two types of data. Primary survey data were collected through face-to-face interviews and self-administered questionnaires. Interviewers received standardized training to reduce response bias. The survey collected data on SWB, satisfaction with public services, livelihood and income-structure characteristics, health information and health-management items, physical activity, sleep, stress-management practices, use of digital health tools, daily-life service use, and individual and household attributes. Secondary data were obtained from official provincial and county sources to measure objective DPS indicators and regional development. Digital infrastructure data were collected from the 2025 statistical yearbooks and annual reports of the health and education commissions of Anhui, Hubei, and Henan provinces, which primarily reflect conditions in 2024. Regional economic data were obtained from county economic yearbooks and statistical bulletins. Education, medical, government-service, e-commerce, express-delivery, and meal-ordering coverage indicators were obtained from the corresponding statistical yearbooks and administrative reports.

### 3.3 Variable selection and descriptive statistics

#### 3.3.1 Dependent variable: rural residents’ SWB

The dependent variable SWB is measured as a continuous composite index ranging from 0 to 1. Its construction is guided by the tripartite conceptualization of subjective well-being—life satisfaction, positive affect, and negative affect—described by Diener et al. (46), with individual items adapted to the rural Chinese context. The index includes 13 items covering these three dimensions. The entropy weight method is used to aggregate these items into a single index. It objectively assigns weights based on data distribution, avoiding the subjectivity inherent in expert weighting, and it gives higher weights to indicators with greater discriminative power, which is particularly important for measuring well-being in heterogeneous rural populations where certain dimensions may be more salient than others. Negative emotion indicators are reverse-coded to ensure consistency. Rural-specific items such as satisfaction with the ecological environment and social security are included to reflect local livelihood conditions. The specific indicator system and measurement standards are shown in Table 2.

**Table 2:** Indicator system for rural residents’ SWB.

Primary dimension	Secondary indicator	Measurement
Life Satisfaction	Health satisfaction	1, Extremely dissatisfied, 2, Dissatisfied, 3, Neutral, 4, Satisfied, 5, Extremely satisfied
	Neighbor relationship satisfaction	Same as above
	Family economic status satisfaction	Same as above
	Work income reasonableness evaluation	1, Extremely unreasonable, 2, Unreasonable, 3, Reasonable, 4, Extremely reasonable
	Family life harmony satisfaction	Same as life satisfaction standards
	Main job satisfaction	Same as life satisfaction standards
	Social security satisfaction	Same as life satisfaction standards
	Ecological environment satisfaction	Same as life satisfaction standards
Positive Emotions	Frequency of feeling calm	1, Never, 2, Rarely, 3, Sometimes, 4, Often, 5, Always
	Frequency of feeling energetic	Same as above
	Feeling of confidence	1, Not at all, 2, Slightly, 3, Quite, 4, Completely
Negative Emotions	Frequency of feeling depressed	1, Never, 2, Rarely, 3, Sometimes, 4, Often, 5, Always (reverse-coded to positive)
	Frequency of physical/mental tiredness	Same as above (reverse-coded)

SWB, subjective well-being. Negative-emotion items are reverse-coded so that higher values indicate better well-being.

The SWB index was constructed using standardized procedures to ensure methodological rigor and reproducibility. All indicators were normalized using the min-max method to eliminate scale differences, with positive indicators transformed as $x_{ij}^{\prime }=(x_{ij}-\min\left(x_{j}\right)/\max\left(x_{j}\right)-\min\left(x_{i}\right)$ and negative indicators using the reversed formula. The entropy weight method was used to assign weights, which objectively reflect the discriminative power of each indicator through the following steps: calculating the proportion of each observation in each indicator as $p_{ij}=x_{ij}^{\prime }/\overset{n}{\underset{i=1}{\sum }}x_{ij}^{\prime }$ , computing the entropy value of each indicator as $(e_{j}=-k\overset{n}{\underset{i=1}{\sum }}p_{ij}\ln\left(p_{ij}\right)$ where k=1/ln(n) , and deriving the final weight of each indicator as $w_{j}=\left(1-e_{j}\right)/\overset{m}{\underset{j=1}{\sum }}\left(1-e_{j}\right)$ . Finally, the standardized indicators were linearly weighted to calculate the final SWB composite index.

The reliability and validity of the SWB index were rigorously tested to ensure measurement quality. The Cronbach’s *α* coefficient for the 13-item SWB scale was 0.87, well above the commonly accepted threshold of 0.7, indicating excellent internal consistency, with sub-dimension coefficients of 0.82 for life satisfaction, 0.79 for positive emotions, and 0.76 for negative emotions. Construct validity was confirmed through exploratory factor analysis, which extracted three factors with eigenvalues greater than 1 explaining 68.4% of the total variance, with all item factor loadings above 0.6 and no cross-loadings exceeding 0.3, supporting the hypothesized three-dimensional structure. A confirmatory factor analysis further demonstrated good model fit, with $\chi^{²}/df=2.34$ , CFI=0.94 , TLI=0.92 , and RMSEA=0.05 . Harman’s single-factor test was conducted to assess common method bias. The unrotated first factor accounted for 32.7% of the total variance, which is below the 40% threshold, suggesting that no single factor dominated the covariance structure.

#### 3.3.2 Core independent variable: DPS

The core independent variable DPS is constructed as a comprehensive index ranging from 0 to 1. It captures the integrated delivery of digital technology and public services across four dimensions: digital education, digital medical care, digital government services, and digital life services. The index combines objective supply data and subjective experience evaluations to avoid one-sided measurement (17, 21). The digital medical-care dimension includes telemedicine and digital health-management service coverage. The digital life-services dimension captures rural e-commerce, express-delivery and meal-ordering service coverage, and respondents’ use of digital payment and other daily-life services. The specific indicator system is shown in Table 3.

**Table 3:** Indicator system for DPS index.

Primary dimension	Secondary indicator	Measurement	Entropy weight	Total dimension weight
Digital education	Online course coverage rate in rural schools	Direct statistical value (e.g., 85% is converted to 0.85)	0.112	0.215
	Satisfaction with digital education	1, Extremely dissatisfied to 5, Extremely satisfied	0.103	
Digital medical care	Telemedicine equipment coverage rate in village clinics	Direct statistical value	0.121	0.276
	Satisfaction with remote consultation	1, Extremely dissatisfied to 5, Extremely satisfied	0.105	
	Digital health-management service coverage	Direct statistical value	0.050	
Digital government	County-level online government service coverage rate	Direct statistical value	0.092	0.184
	Convenience of online certificate handling	1, Extremely inconvenient to 5, Extremely convenient	0.092	
Digital Life services	Rural e-commerce service station coverage rate	Direct statistical value	0.081	0.325
	Express-delivery and meal-ordering service coverage	Direct statistical value	0.068	
	Use of digital payment and other daily-life services	1, Never use to 5, Daily use (standardized to 0–1)	0.176	

DPS, digital public services. All indicators are normalized before entropy weighting. Dimension weights equal the sum of the indicator weights within each DPS dimension.

Missing values, which accounted for less than 2% of all observations, were imputed using the mean value of the corresponding county, and a sensitivity analysis excluding observations with missing values yielded consistent results. Pearson correlation coefficients between all pairs of indicators ranged from 0.23 to 0.67, indicating moderate positive correlations and no serious multicollinearity issues, as confirmed by variance inflation factors all below 3. To test the robustness of the index, we reconstructed it using factor analysis and equal weighting, obtaining correlation coefficients of 0.92 and 0.87, respectively, with the baseline entropy-weighted index, suggesting limited sensitivity of the index to the weighting method.

The weight results show that digital life services (32.5%) and digital medical care (27.6%) are the largest contributing dimensions of the DPS index, reflecting rural residents’ demand for daily convenience, purchasing and delivery access, and medical services. Digital government services have the lowest dimension weight (18.4%), consistent with their comparatively limited penetration in mountainous rural areas.

#### 3.3.3 Control variables

To reduce confounding, the baseline models include individual, household, and county-level covariates identified in prior research (4, 43). Individual covariates include gender, age group, education level, and marital status. Household-level covariates include per capita household income, socioeconomic status, and perceived social fairness. County-level covariates include per capita GDP, transportation infrastructure density, education and healthcare resource supply, and agricultural production conditions.

Regional-level control variables include county-level per capita GDP, transportation infrastructure density, education and healthcare resource supply, and agricultural production conditions. County-level per capita GDP, a continuous variable measuring regional economic development, is denominated in 10,000 yuan and sourced from county-level statistical yearbooks. Transportation infrastructure density is calculated as the total length of highways and rural roads per square kilometer in each county. Education and healthcare resource supply is a composite index averaged from the number of primary and secondary school teachers per 1,000 residents and the number of licensed physicians per 1,000 residents. Agricultural production conditions are measured by the proportion of irrigated arable land in each county, reflecting local agricultural productivity and rural living costs.

#### 3.3.4 Mediating variables

Five mediating variables are constructed to capture potential indirect pathways.

M1 (livelihood stability and income diversification) is a composite index reflecting the structural quality of the household economy, constructed from non-farm income share, number of income sources, and self-rated income stability using the entropy weight method. Higher values indicate more resilient and diversified household livelihoods. M2 (education and medical service quality) captures respondents’ evaluations of local educational and medical service provision. The M2 items assess the overall quality of local education and medical services and are distinct from the DPS indicators measuring satisfaction with digital education and remote consultation. M3 (cultural and social interaction) is measured by the frequency of participation in leisure, neighborhood, and community activities; M4 (daily-life purchasing and delivery convenience) reflects respondents’ perceived convenience in using online shopping, express delivery, meal ordering, digital payment, and online government-service transactions, including transactions involving everyday goods and food purchases; M5 (integrated health and lifestyle behaviors) is a composite index combining health-information use, health self-management, physical activity, sleep quality, stress-management practices, and digital health-tool use. For all composite mediators (M1–M5), component items were standardized and aggregated using the entropy weight method. The component measures were operationalized as follows. Non-farm income share was calculated as the proportion of total household income derived from non-farm sources; the number of income sources ranged from 1 to 5, covering agricultural production, off-farm employment, local business, transfer income, and property income; and self-rated income stability was measured on a 5-point Likert scale (1 = very unstable, 5 = very stable). Education and medical service quality was measured using 4 items rated from 1 (very dissatisfied) to 5 (very satisfied), capturing overall satisfaction with local education quality, education accessibility, medical service quality, and medical accessibility. Cultural and social interaction was assessed using 3 frequency items rated from 1 (never) to 5 (very often), reflecting participation in cultural activities, neighborhood communication, and community collective activities. Daily-life purchasing and delivery convenience was measured using five items assessing online shopping, express delivery, meal ordering, digital payment, and online government service transactions on a 5-point convenience scale ranging from 1 (very inconvenient) to 5 (very convenient). Integrated health and lifestyle behaviors comprised 6 items covering health-information use, health self-management, physical activity, stress-management practices, sleep quality, and digital health-tool use. The five frequency-based items—health-information use, health self-management, physical activity, stress-management practices, and digital health-tool use—were measured on a 5-point frequency scale ranging from 1 (never) to 5 (almost every day); sleep quality was rated separately on a 5-point Likert scale ranging from 1 (very poor) to 5 (very good). All components were oriented so that higher values indicated more favorable conditions.

Table 4 presents descriptive statistics for SWB, DPS, and the control variables in the analytic sample of 1,287 respondents. The mean SWB value is 0.721, suggesting a relatively high overall level of well-being among rural residents in the Dabie Mountains Region. The mean DPS value is 0.449, indicating that digital public services are expanding but have not reached full coverage. Men account for 50.1% of respondents.

**Table 4:** Descriptive statistics.

Variable Name	Symbol	Mean	St. Dev	Min	Max
Subjective well-being	SWB	0.721	0.264	0.208	0.891
Digital public service index	DPS	0.449	0.185	0.152	0.896
Gender	Sex	0.501	0.500	0	1
Age	Age	2.81	1.31	1	5
Education level	Edu	2.89	1.216	1	5
Marital status	Marry	0.813	0.390	0	1
*Per Capita* household income	Income	2.322	4.312	0	19
Socioeconomic status	Socio	1.615	0.495	1	3
County-level *Per Capita* GDP	GDP	5.471	3.74	1.97	9.05
Sense of social fairness	Fair	2.174	1.021	1	5
Transportation infrastructure density	Trans	0.782	0.315	0.214	1.527
Education and healthcare resource supply	EduHealth	0.426	0.183	0.121	0.795
Agricultural Production Conditions	Agri	0.543	0.227	0.189	0.872

Gender is coded 1 for male and 0 for female, and marital status is coded 1 for married or cohabiting respondents and 0 otherwise. Age was coded as 1, 18–30 years; 2, 31–40 years; 3, 41–50 years; 4, 51–60 years; and 5, 61–65 years. Education was coded as 1, primary school or below; 2, junior middle school; 3, senior secondary school/vocational school; 4, junior college; and 5, bachelor’s degree or above. Socioeconomic status and perceived social fairness are treated as ordered variables according to their respective coding schemes.

## 4 Empirical analyses

This section presents empirical examinations of the relationship between DPS and SWB among rural residents in the Dabie Mountains Region. The analysis uses 1,287 observations and proceeds in five steps: baseline regression, robustness checks, instrumental-variable sensitivity analysis, heterogeneity analysis, and exploratory mechanism testing.

### 4.1 Baseline regression results

This section examines Hypothesis 1 by using ordinary least squares (OLS) to estimate the baseline association between DPS and SWB. A multicollinearity test was performed before regression. All variance inflation factors are below 10, suggesting no serious multicollinearity. Hierarchical regressions are conducted by sequentially adding sets of control variables. All regressions employ county-level clustered standard errors to adjust for potential intra-county correlation in unobserved shocks. For the OLS and 2SLS models, the reported t-statistics are based on county-clustered standard errors; the Ordered Probit model reports z-statistics. Results are reported in Table 5.

**Table 5:** Baseline regression results.

Variable	(1)	(2)	(3)	(4)
DPS	0.186^***^ (7.93)	0.164^***^ (6.19)	0.146^***^ (6.13)	0.131^***^ (5.72)
Sex		0.051 (1.19)	0.048 (1.12)	0.044 (1.13)
Age		−0.152^***^ (−5.19)	−0.148^***^ (−5.02)	−0.145^***^ (−4.89)
Edu		0.032 (1.02)	0.030^*^ (1.97)	0.036^*^ (1.89)
Marry		0.082^*^ (1.83)	0.079^*^ (1.76)	0.077^*^ (1.76)
Income			0.091^**^ (2.45)	0.090^**^ (2.42)
Socio			0.112^***^ (3.21)	0.110^***^ (3.19)
Fair			0.235^***^ (4.05)	0.241^***^ (4.20)
GDP				0.024^**^ (2.20)
Trans				0.032^*^ (1.78)
EduHealth				0.067^**^ (2.31)
Agri				0.029 (1.24)
Constant	0.521^***^ (12.36)	0.684^***^ (10.25)	0.592^***^ (8.76)	0.417^***^ (6.32)
*R* ^2^	0.126	0.188	0.214	0.244
*N*	1,287	1,287	1,287	1,287

DPS, digital public services; SWB, subjective well-being; Sex, gender; Age, age group; Edu, education level; Marry, marital status; Income, per capita household income; Socio, socioeconomic status; Fair, perceived social fairness; GDP, county-level per capita gross domestic product; Trans, transportation infrastructure density; EduHealth, education and healthcare resource supply; Agri, agricultural production conditions; *R*^2^, coefficient of determination; *N*, number of observations. Standard errors are clustered at the county level; *t*-statistics are reported in parentheses. *p < 0.1, **p < 0.05, ***p < 0.01.

Table 5 shows the OLS baseline estimates. The regressions examine the direct association between DPS and SWB while sequentially controlling for individual characteristics, household characteristics, and regional economic conditions.

In Column (1), which includes only DPS, the coefficient of DPS is 0.186 and statistically significant at the 1% level. A one-unit increase in DPS is associated with a 0.186-unit increase in SWB, indicating a positive baseline relationship. In the Dabie Mountains Region, integrated digital infrastructure and service platforms expand access to medical consultations, education, agricultural information, and community activities, while inclusive designs may lower usage barriers for vulnerable residents (22, 47). These service functions provide plausible context for the observed positive association.

In Model (2), individual characteristics including gender, age, education, and marital status are added. The coefficient of DPS decreases to 0.164 and remains significant at the 1% level. The modest change indicates that the positive DPS-SWB association remains after adjustment for these individual characteristics.

In Model (3), household variables including per capita household income, socioeconomic status, and perceived social fairness are included. The coefficient of DPS declines to 0.146 but remains statistically significant. Per capita income is also positively associated with SWB, while the adjusted DPS coefficient remains positive.

In Model (4), county-level per capita GDP, transportation infrastructure density, education and healthcare resource supply, and agricultural production conditions are added. The DPS coefficient is 0.131 and remains statistically significant at the 1% level. Transportation infrastructure density and education and healthcare resource supply are also positively associated with SWB.

The control-variable estimates in Model (4) are broadly consistent with rural livelihood conditions. Age is negatively associated with SWB, whereas married respondents report higher SWB. Education has a weak positive coefficient, gender is not statistically significant, and perceived social fairness is positively associated with SWB.

Overall, the results support Hypothesis 1. The positive DPS-SWB association remains statistically significant and stable after adjustment for individual, household, and regional characteristics.

### 4.2 Robustness checks

This section uses four robustness checks to assess the stability of the baseline association. The tests address variable measurement, outlier sensitivity, and model specification. Results are summarized in Table 6.

**Table 6:** Robustness-check results.

Variable	Alternative DPS (factor analysis)	Single-item SWB	1% Winsorization	Ordered probit coefficient
	(1)	(2)	(3)	(4)
DPS	0.126^***^ (4.21)	0.108^***^ (5.27)	0.132^***^ (5.64)	0.088^***^ (3.81)
Control variables	Yes	Yes	Yes	Yes
*R*^2^/Pseudo-*R*^2^	0.233	0.221	0.243	0.199
*N*	1,287	1,287	1,287	1,287

DPS, digital public services; SWB, subjective well-being; *R*^2^, coefficient of determination; *N*, number of observations. Standard errors are clustered at the county level; t-statistics are reported for OLS models and z-statistics for the Ordered Probit model. ***p < 0.01. Column (4) reports pseudo-*R*^2^.

The first robustness check reconstructs the DPS index using factor analysis instead of the entropy weight method. Factor analysis provides an alternative data-driven weighting scheme based on the common variance among the DPS indicators. This robustness check assesses whether the baseline association is sensitive to the weighting and aggregation method used to construct the DPS index. As presented in Column (1) of Table 6, the coefficient remains significantly positive and consistent in direction with the baseline estimate.

The second test adopts an alternative measure of the dependent variable to check for potential bias related to the comprehensive entropy-weighted SWB index. In this test, SWB is measured using a single-item subjective evaluation. Respondents rate their overall well-being on a scale from 1 to 5, where 1 represents extremely unhappy and 5 represents extremely happy. This simplified indicator avoids potential aggregation bias associated with multi-dimensional SWB construction and follows common practice in brief well-being surveys. As shown in Column (2) of Table 6, the coefficient of DPS is 0.108. The coefficient is positive and statistically significant, and it remains consistent with the baseline result. This finding supports the conclusion that the core relationship between DPS and SWB is robust to the use of a more parsimonious SWB measure.

Another robustness test addresses potential distortions caused by extreme values. The SWB variable is winsorized at the 1st and 99th percentiles to reduce the influence of outliers. Extreme values may emerge from temporary emotional fluctuations and could bias standard OLS estimates. Column (3) shows that the DPS coefficient is 0.132, which is nearly identical to the baseline estimate. This result indicates that the observed relationship is not sensitive to the presence of extreme observations.

The final robustness check estimates an Ordered Probit model after dividing the continuous SWB index into five ordered categories using quintile cutoffs. Column (4) reports the Ordered Probit coefficient for DPS, which remains positive and statistically significant.

All four robustness checks support the finding that DPS is stably and positively associated with SWB. Across these alternative specifications, the DPS coefficient remains positive and statistically significant, suggesting that the baseline association is reasonably robust to the examined measurement, outlier, and model choices.

### 4.3 Instrumental-variable sensitivity analysis

Baseline OLS estimates may be influenced by reverse association and omitted variables. Residents with higher SWB may evaluate local DPS more favorably, while unobserved local factors may be associated with both DPS and SWB. As a complementary sensitivity analysis, this study uses two-stage least squares (2SLS) with a geographic instrumental variable.

The instrumental variable is average county-level terrain slope in the Dabie Mountains Region (48). Its relevance is based on the higher construction and maintenance costs of digital infrastructure in steeper terrain. To reduce the possibility that terrain captures other pathways to well-being, the models control for county-level GDP, transportation infrastructure density, education and healthcare resource supply, agricultural production conditions, and individual and household characteristics. The first-stage coefficient and clustered t-statistic are reported in Table 7. Because terrain may still be related to unobserved historical, settlement, or migration characteristics, the exclusion restriction cannot be verified directly; the 2SLS results are therefore interpreted as complementary sensitivity evidence rather than definitive causal estimates.

**Table 7:** Instrumental-variable sensitivity analysis results.

Variable	Stage 1: DPS (1)	Stage 2: SWB (2)
Average county-level terrain slope IV	−0.321^***^ (−13.59)	
Predicted DPS		0.211^***^ (4.24)
Control variables	Yes	Yes
Kleibergen-Paap rk Wald *F* Statistic	184.7	
*R* ^2^	0.231	0.318
*N*	1,287	1,287

DPS, digital public services; SWB, subjective well-being; *R*^2^, coefficient of determination; *N*, number of observations; IV, instrumental variable; 2SLS, two-stage least squares. Standard errors are clustered at the county level; *t*-statistics are reported in parentheses. The model is exactly identified, so an overidentification test is not available. ***p < 0.01.

The 2SLS procedure includes two stages. In the first stage, DPS is regressed on terrain slope and all control variables. As shown in Column (1) of Table 7, the first-stage coefficient for average county-level terrain slope is −0.321 and statistically significant at the 1% level (t = −13.59, clustered at the county level), indicating a strong negative first-stage association: counties with steeper terrain tend to have lower levels of digital public services. The Kleibergen–Paap rk Wald F statistic is 184.7, which is above the conventional rule-of-thumb threshold of 10 and suggests that weak-instrument concerns are limited.

In the second stage, the predicted value of DPS is used to estimate SWB. Column (2) of Table 7 shows a positive and statistically significant coefficient of 0.211. The estimate is larger than the baseline OLS coefficient, which may reflect measurement error, reverse association, or the local nature of the IV estimate. Because the exclusion restriction cannot be tested directly, the result is interpreted as consistent with the positive DPS-SWB association rather than as definitive causal evidence.

### 4.4 Heterogeneity analysis

Baseline results show a positive association between DPS and SWB. This association may differ across DPS dimensions and population groups. Exploring heterogeneity helps identify service components and population groups for which the association is stronger. This section examines coefficient patterns related to Hypotheses 3a–3d using DPS subdimension models and resident-group subsamples. Because formal cross-model and cross-group coefficient-equality tests are not reported, differences in coefficient magnitude are interpreted descriptively. Results are reported in Tables 8, 9.

**Table 8:** Regression results by DPS sub-dimensions.

Variable	DPS Edu (1)	DPS Med (2)	DPS Gov (3)	DPS Life (4)
Sub-dimension index	0.187^***^ (4.89)	0.209^***^ (5.17)	0.109^***^ (3.54)	0.102^***^ (3.32)
Control variables	Yes	Yes	Yes	Yes
*R* ^2^	0.221	0.233	0.208	0.210
N	1,287	1,287	1,287	1,287

DPS Edu, digital education; DPS Med, digital medical care; DPS Gov, digital government services; DPS Life, digital life services. Standard errors are clustered at the county level; *t*-statistics are reported in parentheses. Coefficient magnitudes are compared descriptively; formal equality tests across models are not reported. ***p < 0.01.

**Table 9:** Heterogeneity regression results by resident groups.

Variable	Regional			Income			Education	
	Anhui	Hubei	Henan	Low	Middle	High	Low education	High education
DPS	0.161^***^	0.135^***^	0.103^***^	0.174^***^	0.126^**^	0.080	0.157^***^	0.112^**^
	(4.03)	(2.96)	(2.67)	(4.22)	(2.79)	(1.62)	(4.03)	(2.32)
Control variables	Yes	Yes	Yes	Yes	Yes	Yes	Yes	Yes
*R* ^2^	0.252	0.231	0.218	0.239	0.227	0.205	0.241	0.219
N	410	431	446	532	418	337	784	503

Low-, middle-, and high-income groups were defined using empirical tertile cut points of 1.2 and 2.6 10,000 RMB per capita. All observations tied at the 1.2 10,000 cut-off were assigned to the low-income group; no tied values existed at the 2.6 threshold, leading to unequal sample sizes across income subgroups. Low education corresponds to junior middle school or below (coding 1–2), and high education corresponds to senior secondary education or above (coding 3–5). Standard errors are clustered at the county level; t-statistics are reported in parentheses. **p < 0.05, ***p < 0.01.

#### 4.4.1 Heterogeneity by DPS sub-dimensions

DPS include multiple functional domains. Contributions to SWB may vary according to resident needs. This study divides DPS into four sub-dimensions: digital education (DPS Edu), digital medical care (DPS Med), digital government services (DPS Gov), and digital life services (DPS Life). Regressions use the same control variables as the baseline model.

Table 8 shows that all four DPS subdimensions are positively associated with SWB. Digital medical care has the largest coefficient, followed by digital education, digital government services, and digital life services. This pattern is consistent with the priority needs of rural residents in mountainous areas, where medical and educational resources remain comparatively scarce (8). Digital government and life services are also positively associated with SWB, although their coefficients are smaller. These coefficient patterns are consistent with Hypothesis 3a and with the proposition that services addressing basic health, safety, and human-capital needs may have stronger well-being associations in resource-constrained settings.

#### 4.4.2 Heterogeneity by resident groups

Heterogeneity in the DPS-SWB relationship arises from regional resource conditions, household economic status, and individual human capital. These factors shape access to and use of DPS. This study divides the sample along three dimensions: provincial section (Anhui, Hubei, Henan), household income (low, middle, high), and education level (low, high). Household-income groups were defined using empirical tertile cut points of 1.2 and 2.6 10,000 RMB per capita, derived from the sample distribution of per capita household income. The exact classification rules used in the statistical analysis were: low income, per capita household income ≤ 1.2 10,000 RMB; middle income, 1.2 < per capita household income ≤ 2.6 10,000 RMB; and high income, per capita household income > 2.6 10,000 RMB. Observations tied at the lower cut point were all retained in the low-income group, resulting in clearly unequal subgroup sizes. The education subgroups were defined by educational attainment: respondents with junior middle school education or below (coding 1–2) formed the low-education group, and those with senior secondary education or above (coding 3–5) formed the high-education group. Regression results are reported in Table 9.

Table 9 shows positive and statistically significant within-group DPS coefficients in the three provincial subsamples. The estimated coefficient is largest in Anhui, followed by Hubei and Henan. Because formal cross-group coefficient-equality tests are not reported, these differences are interpreted as descriptive patterns rather than statistically established differences between provinces. The pattern is nevertheless consistent with Hypothesis 3b and with the infrastructure endowment perspective: stronger infrastructure penetration and institutional support may enable broader use of digital public services, while scattered settlements and weaker service outlets may constrain use in other sections of the region (8, 18).

Income heterogeneity may reflect differences in livelihood constraints and access to alternative services. The estimated DPS coefficient is largest in the low-income group, smaller in the middle-income group, and not statistically significant at conventional levels in the high-income group. These within-group estimates are descriptively consistent with Hypothesis 3c and with diminishing marginal utility: lower-income households may place greater value on low-cost digital medical, education, employment, and purchasing services, whereas higher-income households have greater access to private alternatives (43, 47, 49). Formal cross-group equality tests would be required to establish statistically significant differences between income groups.

Education heterogeneity may reflect differences in digital literacy, service expectations, and usage barriers. The estimated DPS coefficient is larger in the lower-education subgroup than in the higher-education subgroup, and both within-group coefficients are positive. This descriptive pattern is consistent with Hypothesis 3d and with the possibility that dialect voice navigation, simplified interfaces, and village digital assistants reduce usage barriers for residents with lower education (24). At the same time, residents with higher education may have more diverse service expectations. Because no formal equality test is reported, the subgroup coefficients should not be interpreted as proof of a statistically significant education-group difference.

### 4.5 Exploratory pathway analysis

This section tests Hypothesis 2 by examining five potential indirect pathways: livelihood stability and income diversification (M1), education and medical service quality (M2), cultural and social interaction (M3), daily-life purchasing and delivery convenience (M4), and integrated health and lifestyle behaviors (M5). All pathways are estimated using the exploratory two-step OLS mediation framework specified in Equations 2, 3, with the full set of baseline control variables included in each model for consistency with the baseline specification.

The construction of M1–M5 is described in Section 3.3.4. Table 10 reports a separate two-step model for each mediator using the same control variables as the baseline specification. To minimize construct overlap, M2 uses evaluations of overall local education and medical service quality rather than the digital-service satisfaction items included in the DPS index. M4 uses perceived accessibility and time-saving evaluations rather than the county-level coverage and service-use frequency indicators included in the DPS index. No identical item is used in both the predictor and mediator constructs.

**Table 10:** Exploratory indirect-pathway analysis results.

Variable	Livelihood stability and income diversification (M1)		Education and medical service quality (M2)		Cultural and social interaction (M3)		Daily-life purchasing and delivery convenience (M4)		Integrated health and lifestyle behaviors (M5)	
	Mediator	SWB	Mediator	SWB	Mediator	SWB	Mediator	SWB	Mediator	SWB
	(1)	(2)	(3)	(4)	(5)	(6)	(7)	(8)	(9)	(10)
DPS	0.097^***^ (4.32)	0.113^***^ (4.06)	0.105^***^ (4.49)	0.111^***^ (3.90)	0.061^***^ (3.86)	0.108^***^ (4.40)	0.073^**^ (2.26)	0.124^***^ (4.90)	0.085^***^ (3.72)	0.118^***^ (4.65)
Mediator		0.215^***^ (4.62)		0.192^***^ (3.91)		0.435^**^ (2.33)		0.126^*^ (1.72)		0.193^***^ (4.12)
Control variables	Yes	Yes	Yes	Yes	Yes	Yes	Yes	Yes	Yes	Yes
Indirect association		0.0211		0.0202		0.0262		0.0091		0.0164
95% BCa CI	[0.0115,	0.0324]	[0.0098,	0.0327]	[0.0135,	0.0416]	[0.0012,	0.0187]	[0.0072,	0.0279]
*R* ^2^	0.280	0.261	0.295	0.291	0.259	0.248	0.213	0.238	0.254	0.272
*N*	1,287	1,287	1,287	1,287	1,287	1,287	1,287	1,287	1,287	1,287

DPS, digital public services; SWB, subjective well-being; BCa, bias-corrected and accelerated bootstrap; CI, confidence interval. Indirect associations are calculated using the coefficient-product method. Each mediator is estimated in a separate exploratory model; the estimates may represent overlapping pathways and should not be interpreted as additive. BCa confidence intervals are based on 5,000 bootstrap replications. *p < 0.1, **p < 0.05, ***p < 0.01.

The mediators represent distinct economic, service-quality, social, convenience, and health-behavior domains, whereas SWB captures respondents’ overall life evaluation and affective experience.

Pearson correlation coefficients between each mediator and the overall SWB index are 0.324 for M1, 0.357 for M2, 0.412 for M3, 0.289 for M4, and 0.376 for M5. These moderate correlations indicate that the mediators are meaningfully associated with yet empirically distinct from SWB. The pathway estimation results are reported in Table 10.

In Table 10, the M1 index of livelihood stability and income diversification reflects the structural resilience of household economic conditions associated with DPS. The Dabie Mountains Region faces high production risks, limited market access, and volatile agricultural income. Digital platforms may expand non-farm employment channels, diversify income sources, improve access to employment and market information, and support more stable production and operational decisions (27). DPS is positively associated with more stable and diversified livelihoods, and this mediator remains positively associated with SWB after controlling for per capita household income and other covariates. The results are consistent with livelihood resilience as one potential indirect pathway linking DPS to SWB (29).

Education and medical service quality (M2) captures access to and evaluations of local education and medical services. Rural areas have long faced shortages of teachers and doctors (4). Digital platforms provide online courses and telemedicine services that connect rural residents with wider service resources (23). DPS is positively associated with education and medical quality, and this mediator is positively associated with SWB in the adjusted model. These estimates are consistent with service quality as a second potential indirect pathway.

Cultural and social interaction (M3) measures the frequency of participation in leisure, neighborhood, and community activities (50). Digital services may provide additional channels for cultural participation, information exchange, and neighborhood interaction, particularly in geographically dispersed communities. Scattered settlements and out-migration can constrain cultural supply and social connections in mountainous regions (26, 35). DPS is positively associated with cultural and social interaction, which is in turn positively associated with SWB. The estimates support social connection as a third potential indirect pathway.

Daily-life purchasing and delivery convenience (M4) captures perceived transaction efficiency and service accessibility across online shopping, express delivery, meal ordering, digital payment, and online government-service transactions. DPS is positively associated with M4, while the association between M4 and SWB is positive at the 10% significance level. These estimates are consistent with purchasing and delivery convenience as a comparatively modest fourth indirect pathway (34, 38).

Integrated health and lifestyle behaviors (M5) capture health-information use, health self-management, physical activity, sleep quality, stress-management practices, and digital health-tool use. DPS is positively associated with M5, and M5 is positively associated with SWB after adjustment for DPS and the control variables. The estimated indirect association is 0.0164, with a 95% BCa bootstrap confidence interval of [0.0072, 0.0279]. This result supports integrated health and complementary lifestyle behaviors as one potential pathway linking digital services to well-being.

Bootstrap inference based on 5,000 replications produced confidence intervals that excluded zero for all five estimated indirect associations. Because the mediators were estimated in separate models and may represent overlapping pathways, the indirect associations should not be interpreted as additive or directly comparable shares of a single total association. The results provide exploratory support for Hypothesis 2, subject to the temporal limitations of cross-sectional mediation analysis.

## 5 Discussion, conclusions and recommendations

### 5.1 Discussion

Building on the empirical evidence presented in the preceding sections, this discussion situates the associations between DPS and SWB in the cross-provincial Dabie Mountains Region within broader theoretical frameworks, contextualizes the findings against existing domestic and international scholarship, and outlines key limitations alongside directions for future research.

#### 5.1.1 Theoretical extensions

These empirical findings carry three distinct theoretical implications that refine established frameworks for understanding digital welfare associations in geographically fragmented, multi-jurisdictional settings.

First, the findings add nuance to the infrastructure endowment perspective. Aggregate infrastructure coverage alone may not explain variation in the well-being associations of digital public services across regions with comparable headline penetration rates. The cross-provincial coefficient pattern suggests two relevant boundary conditions: the intensity of intergovernmental policy support and the depth of infrastructure penetration in dispersed rural settlements. In multi-jurisdictional mountainous regions, administrative fragmentation and scattered settlement patterns may weaken the relationship between nominal coverage and effective service use. Future research should therefore supplement aggregate coverage metrics with measures of penetration depth, service quality, and institutional coordination.

Second, the findings contribute to the digital-literacy threshold perspective. The conventional framework predicts larger digital benefits at higher levels of educational attainment, whereas the subgroup coefficients in this study show a descriptive “catch-up” pattern among residents with lower education. Inclusive features such as dialect-based navigation, simplified interfaces, and village-level assistance may help explain this pattern. Because formal cross-group equality tests are not reported, the result should be interpreted as suggestive rather than as definitive evidence of a reversal effect. It nevertheless highlights inclusive service design as a potentially important condition shaping who benefits from digital public services.

Third, the findings refine the contextual scope of subjective well-being theory. Existing frameworks, largely developed in urban settings, often emphasize absolute income level as the dominant pathway linking digital services to well-being. In the Dabie Mountains Region, cultural and social interaction has the largest estimated indirect association among the separately modeled pathways, although differences in magnitude should be interpreted descriptively because the mediators were not estimated jointly. Daily-life purchasing and delivery convenience offers a further service-access channel. This pattern suggests that the relative importance of material, social, health, and convenience pathways may vary with settlement density, geographic isolation, and local service constraints.

#### 5.1.2 Comparison with existing literature

The positive DPS–SWB association is consistent with previous research on the welfare potential of rural digital services (6, 23). The present study extends this literature by identifying an integrated health and lifestyle pathway alongside economic, service-quality, social, and daily-life purchasing pathways.

First, the prominence of socio-cultural connection differs from urban-based studies that often emphasize absolute income growth as the principal mechanism. The result is consistent with evidence from remote rural China that social and cultural pathways can be especially relevant in geographically isolated communities (51). This pattern may reflect the importance of unmet social and cultural needs in geographically dispersed communities. Because the pathways were estimated separately using cross-sectional data, their relative ordering should be interpreted as descriptive rather than as evidence that DPS produce a structural shift from material to socio-cultural benefits.

Second, the integrated health and lifestyle pathway is consistent with evidence that physical activity, sleep, stress management, and health self-management operate as complementary rather than isolated behaviors. Digital public services can combine health information, activity support, sleep-management resources, stress-management tools, and social support within a common delivery infrastructure (33, 39). Rural e-commerce, express delivery, and online meal-ordering services provide a separate but complementary access function by reducing the time and distance required for everyday food purchasing in dispersed communities.

Third, comparison with research on the Qinba Mountains suggests that the dominant pathways associated with digital services may differ even across topographically similar regions (7). Infrastructure integration appears especially important where basic coverage remains limited, whereas socio-cultural and lifestyle services may become more salient after basic digital access is established. This comparison supports a context-sensitive and potentially sequential view of mountainous digital rural development.

These findings indicate that integrated digital public services may provide an integrated platform for health management, multidomain lifestyle support, and everyday purchasing access in resource-constrained rural settings. Embedding these functions within routine public-service delivery may improve their reach among low-income and low-education groups. The findings therefore support DPS as a platform for technology-supported health and lifestyle services, while the food-purchasing pathway should be interpreted as an accessibility and convenience mechanism.

Taken together, these comparisons suggest that the well-being associations of digital public services are contingent on local geographic constraints, development stages, and cultural endowments. Universal models may therefore mischaracterize digital-welfare patterns in non-urban settings, underscoring the need for context-specific theoretical and policy frameworks.

#### 5.1.3 Limitations and future research

This study has six key limitations. First, the cross-sectional observational design identifies associations and cannot establish temporal ordering or definitive causality. Panel data would clarify the evolution of digital-service and lifestyle pathways over time. Second, self-reported SWB, service evaluations, and lifestyle behaviors may be affected by common-method or recall bias, although objective county indicators and robustness checks reduce this concern. Third, excluded and unreturned questionnaires did not provide complete analyzable information, so potential nonresponse bias cannot be ruled out. Fourth, evidence from one mountainous region may not generalize to more developed rural areas or other institutional settings. Fifth, because the sampling frame was restricted to adults aged 18–65 years, the findings may not generalize to older rural residents, an important population for health and well-being research. Sixth, geographic IV estimates depend on an exclusion restriction that cannot be verified directly. Future longitudinal studies, natural experiments, and intervention evaluations could provide stronger causal and implementation evidence.

### 5.2 Conclusion

Using micro-survey data from the Dabie Mountains Region spanning Anhui, Hubei, and Henan provinces, this study examines the association between digital public services and rural residents’ subjective well-being, together with heterogeneous patterns and five potential indirect pathways. The analysis yields three main conclusions.

First, higher levels of DPS are consistently associated with higher SWB across the baseline and robustness models. Second, the estimated coefficient is descriptively larger among low-income and less-educated residents and varies descriptively across provincial sections in line with infrastructure and policy conditions. Third, the association is linked to multiple complementary pathways, including socio-cultural connection, livelihood stability and income diversification, education and medical service quality, daily-life purchasing and delivery convenience, and integrated health and lifestyle behaviors.

Overall, the findings indicate that digital public services are positively associated with well-being and may offer comparatively inclusive benefits in geographically disadvantaged rural areas. They support context-specific digital rural policies that combine inclusive service design and cross-jurisdictional coordination with digital health information and self-management support, physical activity, sleep, stress-management functions, and convenient food-purchasing and delivery services.

### 5.3 Recommendations

Based on empirical results from the Dabie Mountains Region covering Anhui, Hubei, and Henan provinces, this study proposes targeted policy suggestions to improve DPS and enhance the SWB of rural residents.

First, differentiated strategies should be implemented across the three provincial sections. For the Anhui section, efforts should focus on high-quality integration of DPS with cultural tourism and characteristic industries to improve service efficiency and added value. For the Hubei section, policies should strengthen the integration of digital services with characteristic agriculture, red tourism, and public health systems and expand coverage of digital education and telemedicine. For the Henan section, priority should be given to improving digital infrastructure, expanding 5G and telemedicine coverage in remote villages, and promoting low-cost and user-friendly services to narrow regional gaps.

Second, policies should prioritize projects that enhance livelihood resilience and stabilize household income. Digital platforms should provide better agricultural information, technical guidance, and online sales channels to improve operational stability and create diversified non-farm employment opportunities. Steady investment should be maintained in digital medical care and digital education to address shortages of high-quality basic public services in mountainous areas.

Third, accessibility and inclusiveness should be improved to benefit low-income and low-education groups. Inclusive designs including dialect voice navigation, large-font interfaces, and simplified procedures should be retained and optimized. Village-level digital assistants should provide on-site guidance for vulnerable groups. Digital literacy training should be extended to remote villages to reduce the digital divide.

Fourth, the five potential pathways identified in the exploratory analyses should be considered in an integrated manner. Digital agriculture and e-commerce can support livelihood stability and income diversification; online classrooms and telemedicine can improve education, medical care, and health self-management. Rural e-commerce stations, express-delivery networks, and online meal-ordering services can also reduce the time and distance required for everyday food purchasing.

Fifth, health and multidomain lifestyle support should be integrated into the DPS system. Digital medical and education platforms can provide locally adapted health guidance, chronic-disease self-management support, physical-activity programs, sleep-management resources, and stress-management tools. Digital life-service platforms can connect remote villages with online shopping, express delivery, and meal-ordering services through existing rural digital-service networks, thereby improving the convenience of everyday food purchasing. Simplified interfaces, dialect-based content, and village digital assistants should be prioritized so that low-income, older, and less-educated residents can use these services effectively.

Finally, cross-provincial coordination should be strengthened among Anhui, Hubei, and Henan. The three provinces should unify infrastructure and service standards, share governance experience and industrial empowerment models, and jointly build demonstration zones for digital countryside development. Coordinated construction can further improve the quality and equity of DPS and support the SWB of rural residents in the entire Dabie Mountains Region.

## Data Availability

The raw data supporting the conclusions of this article will be made available by the authors, without undue reservation.
